# Head-to-head comparison of plasma and PET imaging ATN markers in subjects with cognitive complaints

**DOI:** 10.1186/s40035-023-00365-x

**Published:** 2023-06-29

**Authors:** Jiaying Lu, Xiaoxi Ma, Huiwei Zhang, Zhenxu Xiao, Ming Li, Jie Wu, Zizhao Ju, Li Chen, Li Zheng, Jingjie Ge, Xiaoniu Liang, Weiqi Bao, Ping Wu, Ding Ding, Tzu-Chen Yen, Yihui Guan, Chuantao Zuo, Qianhua Zhao, on behalf of the Shanghai Memory Study (SMS), Keliang Chen, Langfeng Shi, Wanqing Wu, Yan Zhou, Yan Zhang, Fang Pei

**Affiliations:** 1 Department of Nuclear Medicine and PET Center, Huashan Hospital, Fudan University, Shanghai, China; 2 National Clinical Research Center for Aging and Medicine and National Center for Neurological Disorders, Huashan Hospital, Fudan University, Shanghai, China; 3 Department of Neurology, Huashan Hospital, Fudan University, Shanghai, China; 4 Department of Ultrasound, Huashan Hospital, Fudan University, Shanghai, China; 5 APRINOIA Therapeutics Co., Ltd, Suzhou, China; 6 Human Phenome Institute, Fudan University, Shanghai, China; 7 MOE Frontiers Center for Brain Science, Fudan University, Shanghai, China

**Keywords:** ATN biomarkers, PET imaging, Plasma, Clinical severity

## Abstract

**Background:**

Gaining more information about the reciprocal associations between different biomarkers within the ATN (Amyloid/Tau/Neurodegeneration) framework across the Alzheimer’s disease (AD) spectrum is clinically relevant. We aimed to conduct a comprehensive head-to-head comparison of plasma and positron emission tomography (PET) ATN biomarkers in subjects with cognitive complaints.

**Methods:**

A hospital-based cohort of subjects with cognitive complaints with a concurrent blood draw and ATN PET imaging (^18^F-florbetapir for A, ^18^F-Florzolotau for T, and ^18^F-fluorodeoxyglucose [^18^F-FDG] for N) was enrolled (*n* = 137). The β-amyloid (Aβ) status (positive versus negative) and the severity of cognitive impairment served as the main outcome measures for assessing biomarker performances.

**Results:**

Plasma phosphorylated tau 181 (p-tau181) level was found to be associated with PET imaging of ATN biomarkers in the entire cohort. Plasma p-tau181 level and PET standardized uptake value ratios of AT biomarkers showed a similarly excellent diagnostic performance for distinguishing between Aβ+ and Aβ− subjects. An increased tau burden and glucose hypometabolism were significantly associated with the severity of cognitive impairment in Aβ+ subjects. Additionally, glucose hypometabolism – along with elevated plasma neurofilament light chain level – was related to more severe cognitive impairment in Aβ− subjects.

**Conclusion:**

Plasma p-tau181, as well as ^18^F-florbetapir and ^18^F-Florzolotau PET imaging can be considered as interchangeable biomarkers in the assessment of Aβ status in symptomatic stages of AD. ^18^F-Florzolotau and ^18^F-FDG PET imaging could serve as biomarkers for the severity of cognitive impairment. Our findings have implications for establishing a roadmap to identifying the most suitable ATN biomarkers for clinical use.

**Supplementary Information:**

The online version contains supplementary material available at 10.1186/s40035-023-00365-x.

## Introduction

With the increase of the aging population worldwide, cognitive impairment is posing a tremendous burden on our society. In addition to dementia [1], subjective cognitive decline (SCD) [2] and mild cognitive impairment (MCI) [3] are two stages of cognitive decline that frequently occur in advanced age. Alzheimer’s disease (AD), the most common form of dementia, is a multifaceted disease with different pathological and mechanistic substrates. The biological definition of AD, that is, the ATN (Amyloid/Tau/Neurodegeneration) framework, aiming for more precise and early disease identification, has gained substantial attraction in research settings [4]. The ATN biomarkers come in three major forms: cerebrospinal fluid (CSF), plasma, and imaging biomarkers. The development of highly specific immunoassays for CSF and plasma biomarkers and recent advances in the field of positron emission tomography (PET) imaging have largely improved the diagnostic accuracy. Notably, there is accumulating evidence supporting complimentary roles for different sets of biomarkers. For example, a rise of CSF or plasma tau species appears to precede abnormal tau PET imaging during the course of AD [5]. This calls for a better understanding of the reciprocal interrelationships between different biomarker matrices within the ATN framework. There is also an unmet need to standardize and validate a strategic roadmap for routine application of ATN biomarkers in memory clinics [6]. Meanwhile, the assessment of relationship between ATN biomarkers and cognitive symptoms (C) is important given that a clinical-biological rather than a purely biological diagnosis of AD is recommended in clinical settings [7].

In recent years, much has been learned on the diagnostic performances of traditional CSF and imaging A (β-amyloid [Aβ] PET, CSF Aβ_42_, and CSF Aβ_42_/Aβ_40_ ratio), T (tau PET and CSF phosphorylated tau [p-tau]), and N (anatomic magnetic resonance imaging [MRI], ^18^F-fluorodeoxyglucose [^18^F-FDG] PET, and CSF total tau [t-tau]) biomarkers [4, 7]. Although plasma as a potential source of ATN markers has been increasingly explored to reduce the use of invasive lumbar punctures [8], much validation work remains to be done. Regarding the association between fluid and imaging biomarkers, CSF (Aβ_42_ for A, p-tau for T, neurofilament light chain [NfL] for N) and imaging (^18^F-flutemetamol PET for A, ^18^F-flortaucipir PET for T, anatomic MRI for N) biomarkers are reported to be not interchangeable and the optimal approach varies by clinical stage [9]. Recently, plasma p-tau biomarkers (p-tau181, p-tau217, p-tau231) have been suggested to be valid indicators of amyloid and tau PET in clinical and community populations [10–16], although multiple comorbidities may affect the interpretation of these biomarkers [16]. Meanwhile, similar to the non-commutable correlations between CSF and imaging biomarkers, plasma p-tau (p-tau181, p-tau217, p-tau231) and tau PET (^18^F-flortaucipir, ^18^F-RO948, ^18^F-MK6240) biomarkers are thought to reflect different stages of tau pathology progression [17–19].

In this study, we present a comprehensive head-to-head comparison of plasma (Aβ_42_/Aβ_40_ for A, p-tau181 for T, as well as NfL and t-tau for N) and PET imaging (^18^F-florbetapir for A, ^18^F-Florzolotau for T, and ^18^F-FDG for N) ATN biomarkers in a hospital-based cohort of patients with cognitive complaints admitted to a memory clinic. The Aβ status (positive *versus* negative) and the severity of cognitive impairment served as the main outcome measures for assessing biomarker performances. The four plasma biomarkers included in the current study are relatively well-established and more readily available than other newly developed ones. The FDA-approved amyloid radiotracer ^18^F-florbetapir plays a cornerstone role in the diagnosis of AD [20]. The second-generation tau ligand ^18^F-Florzolotau (also known as ^18^F-APN-1607 or ^18^F-PM-PBB3) could overcome the limitations of first-generation tau PET tracers and reduce off-target binding [21]. ^18^F-FDG is the most used PET tracer in nuclear medicine and its accessibility is significantly higher than that of any A and T PET imaging. The current study therefore provides a deeper insight into the comparability of plasma and PET imaging ATN biomarkers, which may be helpful for clinical and research applications. The outstanding strengths of the current study were that all participants were consecutively recruited from a real-life memory clinic, and the aforementioned biomarkers were available to all participants.

## Methods

### Participants

All procedures and visits occurred at the Memory Clinic of the Department of Neurology, Huashan Hospital, Fudan University (Shanghai, China) and the study was conducted as part of the hospital-based Shanghai Memory Study (SMS) [22]. Patients consecutively enrolled in the SMS were considered eligible if they presented with cognitive complaints and agreed on venous blood sampling (*n* = 260). After exclusion of subjects who refused multiple PET examinations due to radiation concerns (*n* = 114), the remaining 146 patients underwent assessment of PET ATN biomarkers using three different tracers (^18^F-florbetapir for A, ^18^F-Florzolotau for T, and ^18^F-FDG for N). After the additional exclusion of patients who had contraindications to structural MRI (*n* = 9), the final population of interest consisted of 137 subjects. The study participants were finally categorized as being either Aβ-positive (Aβ+) or Aβ-negative (Aβ−) as described below. A flowchart of patient recruitment is shown in Additional file 1: Figure S1. Variables collected for all participants included age, sex, years of education, and the presence of at least one apolipoprotein E (*APOE*) ε4 allele.

### Diagnostic criteria

All clinical diagnoses were reached by consensus, following clinical interview and review of neuropsychological and biomarker data. Dementia was diagnosed according to the DSM-IV criteria [23], whereas the diagnosis of MCI was made according to the Petersen’s criteria [3]. When a subject did not meet the criteria for MCI or any dementia but reported subjective experience of cognitive decline on one or more cognitive domains, an SCD label was assigned [24]. The clinical diagnosis of AD was based on the NINCDS-ADRDA criteria [25] along with Aβ PET findings [4]. According to the ATN framework [4], all participants with positive findings in ^18^F-florbetapir amyloid PET (Aβ+) were within the AD spectrum (cognitive impairment due to AD) while those with negative findings (Aβ−) were ruled out from the AD continuum (cognitive impairment not due to AD).

### Evaluation of cognitive impairment severity

Clinical Dementia Rating (CDR) is a semi-structured inventory covering six cognitive, behavioral, and functional aspects. The neurologists need to score each of the above aspects with reference to information collected from participants and proxy. The global CDR score was calculated and the severity of cognitive impairment was assessed using a 5-point scale (0, 0.5, 1.0, 2.0, and 3.0) based on the Washington University CDR-assignment algorithm, with higher levels indicating higher severity [26, 27].

### Neuropsychological testing

The study participants underwent extensive neuropsychological testing to assess global cognition, instrumental activities of daily living, as well as memory, visuospatial, language, attention, and executive functions. Global cognition was assessed using the Mini-Mental State Examination (MMSE) and the Montreal Cognitive Assessment (MOCA). Instrumental activities of daily living were investigated using the Functional Assessment Questionnaire (FAQ) questionnaire. Raw scores of the Auditory Verbal Learning Test, Rey-Osterrieth Complex Fig test, Boston Naming Test, Trail Making Test, Clock Drawing Test, Verbal Fluency Test, Symbol Digit Modalities Test, and Similarity Test were collected and Z-transformed based on previously reported normative data [28]. Z-scores for each test were grouped according to specific cognitive domains (i.e., memory, visuospatial function, language, attention, and executive functions) and averaged for subsequent analyses [28].

### Quantification of plasma ATN biomarkers

Whole blood collected into spray-coated K_2_EDTA tubes was centrifuged at 1000 rpm for 15 min at 4 °C. The plasma fraction was transferred to a new 1.5-ml tube (Eppendorf, Hamburg, Germany) and stored at − 80 °C until use. Plasma levels of Aβ_40_ (A), Aβ_42_ (A), p-tau181 (T), t-tau (N), and NfL (N) were measured simultaneously on a Simoa® HDx analyzer (Quanterix, Billerica, MA), according to the manufacturer’s instructions. The Aβ_42_/Aβ_40_ ratio was subsequently determined and used as the plasma Aβ biomarker (A). Laboratory personnel were blinded to clinical information and imaging data. A detailed protocol has been reported elsewhere [29].

### Image acquisition

The mean (standard deviation) interval from PET imaging to blood collection was 2.6 (5.0) weeks. PET imaging ATN biomarkers were assessed using the following tracers: ^18^F-florbetapir for A (^18^F-AV-45; 50 − 70 min post-injection), ^18^F-Florzolotau for T (90 − 110 min post-injection), and ^18^F-FDG for N (60 − 70 min post-injection). Static images were acquired on different days on a Biograph mCT Flow PET/CT system (Siemens, Erlangen, Germany). High-resolution structural MRI images obtained with a 3.0-T horizontal magnet scanner (Discovery MR750; GE Medical Systems, Milwaukee, WI) were used for spatial normalization. The protocols used for acquisition have been described previously in detail [30–32].

### Assessment and classification of Aβ PET images

Raw Aβ PET images were visually interpreted using dedicated software (Siemens syngo.via) by two independent neuroradiologists (CZ, more than 20 years of experience; JL, more than 5 years of experience) blinded to clinical and laboratory data. Each participant was classified as either Aβ-positive (Aβ+) or Aβ-negative (Aβ−) according to the criteria proposed previously [33]. A third expert (HZ, more than 10 years of experience) was invited to review images in case of discrepancies; the final classification was based on majority voting.

### Image processing and semi-quantitative analysis

Images were processed via Statistical Parametric Mapping 12 (SPM12; http://www.fil.ion.ucl.ac.uk/spm/software/spm12/) implemented in MATLAB (version 2018b, MathWorks, Natick, MA). Prior to smoothing (full-width at half-maximum: 8 mm), raw PET images were first co-registered to the concurrent structural MRI images and then spatially normalized to the Montreal Neurological Institute standard space using the transformation matrices of segmented individual structural MRI images. The following reference regions were selected: whole cerebellum for ^18^F-florbetapir PET [34], cerebellar grey matter for ^18^F-Florzolotau PET [31], and pons for ^18^F-FDG PET [35]. Standardized uptake value ratio (SUVR) images were obtained after reference region-based intensity normalization. Aβ SUVR values were quantified in the following regions of interest (ROIs) defined according to the Automated Anatomical Labelling Atlas three (AAL3) [36]: bilateral frontal lobes, anterior cingulate gyrus, posterior cingulate gyrus, lateral parietal gyrus and lateral temporal gyrus. In accordance with previous methodology [34], the unweighted average SUVR value of the ROIs located above was considered as the global Aβ SUVR. Tau SUVR values were determined in the following ROIs according to the Desikan-Killiany Atlas [37]: bilateral entorhinal cortex, amygdala, middle temporal gyrus, and inferior temporal gyrus. In keeping with a previous study [38], the unweighted average SUVR value of the first two ROIs and the weighted average SUVR value of the last two ROIs were considered as the medial temporal lobe (MTL) and temporal neocortex (NEO-T) SUVR values for tau, respectively. ^18^F-FDG SUVR values were quantified in the following ROIs defined according to the AAL3 [36]: bilateral angular gyri, posterior cingulate gyrus, and inferior temporal gyrus. The unweighted average SUVR value of all ROIs was considered as the meta-analytically derived region of interest (metaROI) SUVR for determining an abnormal glucose metabolic activity [39].

### Statistics

The Kolmogorov–Smirnov test was used to test the normal distribution of continuous variables. Intergroup comparisons between Aβ+ and Aβ− subjects were performed using independent Student’s *t*-tests (normally distributed continuous variables), Mann–Whitney *U* tests (skewed continuous variables), and chi-square tests (categorical variables), as appropriate. The reciprocal associations between plasma and PET imaging ATN biomarkers were investigated at both voxel and region levels. For voxel-wise analysis, the multiple regression model implemented in SPM12 was applied to the entire cohort, as well as separately to Aβ+ and Aβ− subjects. Age, sex, and the interval from PET imaging to blood collection (expressed in weeks) were entered as covariates, with the statistical threshold being set at a family wise error (FWE)-corrected *P* value (*P*_FWE_) < 0.05. For region-level assessments, partial correlation analysis after adjustment for age, sex, and the interval from PET imaging to blood collection was applied to the entire cohort, as well as separately to Aβ+ and Aβ− subjects. Generalized linear models (GLMs) adjusted for age and sex were used to compare the performances of plasma and PET imaging ATN biomarkers in Aβ+ and Aβ− subjects. The ability of biomarkers in distinguishing the Aβ status was determined by receiver operating characteristic (ROC) curve analysis. Agreement and Cohen’s kappa were calculated to assess the concordance between biomarkers. The optimal cutoff values for biomarker-based classification were determined with the greatest Youden’s index based on the ROC curve analysis. The associations between plasma and PET imaging ATN biomarkers and the severity of cognitive impairment (CDR scores) were investigated using GLMs adjusted for age and sex in the entire cohort, as well as separately to Aβ+ and Aβ− subjects. Finally, age- and sex-adjusted partial correction analysis was implemented to investigate the correlations between biomarkers and the results of neuropsychological testing. Data were analyzed using SPSS, version 23 (IBM, Armonk, NY), unless otherwise indicated. Bonferroni-corrected *P* values (*P*_c_) were used to adjust for multiple comparisons. Two-tailed *P* values < 0.05 were considered statistically significant, unless otherwise indicated. Further adjustments for education and *APOE* ε4 were also made for all analysis where applicable and relevant results are presented in Additional file 1: Fig. S2, Fig. S4–S6, Table S1–S4.

## Results

### Participants

The final study cohort consisted of 137 patients with cognitive complaints who were classified as either Aβ-positive (Aβ+; *n* = 90) or Aβ-negative (Aβ−; *n* = 47) based on visual interpretation of ^18^F-florbetapir PET imaging findings. The Aβ+ group comprised 29 patients with MCI due to AD and 61 with AD dementia, whereas the Aβ− group included 10 subjects with SCD, 26 patients with MCI not due to AD and 11 with dementia not due to AD. There were no intergroup differences in terms of age, years of education, and interval from PET imaging to blood collection (Table 1); however, the Aβ+ group showed more severe cognitive impairment and included a higher proportion of women and *APOE* ε4 allele-carries.

**Table 1:** General characteristics of the study participants stratified according to the Aβ status

	Aβ+ subjects	Aβ− subjects	*P* value
Number of subjects	90	47	–
Age, years	65.5 (9.6)	66.1 (7.9)	0.696 ^a^
Sex, female, %	61.1	42.6	0.047 ^b^
Education, years	10.6 (3.9)	11.7 (4.6)	0.165 ^a^
*APOE* ε4 carries, % ^e^	61.8	21.3	< 0.001 ^b^
Interval from PET imaging to blood collection, weeks	2.3 (4.6)	3.2 (5.6)	0.318 ^a^
*Neuropsychological tests*			
CDR, number of subjects	CDR = 0, 0 CDR = 0.5, 29 CDR = 1, 45 CDR = 2, 13 CDR = 3, 3	CDR = 0, 8 CDR = 0.5, 30 CDR = 1, 6 CDR = 2, 3 CDR = 3, 0	< 0.001 ^c^*
MMSE	22.0 [19.0, 25.0]	26.0 [24.0, 28.0]	< 0.001 ^c^*
MOCA ^e^	16.0 [9.5, 19.0]	19.0 [16.0, 24.0]	< 0.001 ^c^*
FAQ	12.5 [8.8, 18.0]	7.0 [4.0, 9.0]	< 0.001 ^c^*
Memory ^e, f^	− 2.1 [− 2.5, − 1.7]	− 1.6 [− 2.1, − 0.6]	< 0.001 ^c^*
Visuospatial function ^e, f^	− 1.7 [− 6.9, 0.1]	0.0 [− 2.4, 0.7]	0.002 ^c^*
Language ^e, f^	− 1.6 [− 2.8, − 0.6]	− 0.9 [− 1.7, − 0.2]	0.004 ^c^*
Attention ^e, f^	− 2.1 [− 6.3, − 0.6]	− 0.7 [− 1.4, 0.0]	< 0.001 ^c^*
Executive function ^e, f^	− 3.8 [− 10.1, − 1.3]	0.1 [− 2.5, 2.5]	< 0.001 ^c^*
*PET ATN biomarkers*			
A: Global SUVR	1.4 [1.3, 1.5]	1.2 [1.1, 1.2]	< 0.001 ^d^*
T: MTL SUVR	1.6 [1.4, 1.8]	1.1 [1.0, 1.2]	< 0.001 ^d^*
T: NEO-T SUVR	1.5 [1.3, 1.8]	1.0 [1.0, 1.1]	< 0.001 ^d^*
N: metaROI SUVR	1.2 [1.1, 1.4]	1.4 [1.4, 1.6]	< 0.001 ^d^*
*Plasma ATN biomarkers*			
A: Aβ_42_/Aβ_40_ ratio	0.04 [0.04, 0.06]	0.05 [0.04, 0.06]	0.054 ^d^
T: P-tau181, pg/ml	4.8 [3.9, 6.0]	2.5 [1.8, 2.9]	< 0.001 ^d^*
N: T-tau, pg/ml	3.4 [2.3, 5.4]	3.2 [2.1, 4.6]	0.238 ^d^
N: NfL, pg/ml	18.0 [14.5, 21.6]	13.6 [8.8, 20.7]	0.064 ^d^

Data are presented as mean (standard deviation) or median [quartile 1, quartile 3], unless otherwise indicated

Each participant was classified as either Aβ-positive (Aβ+) or Aβ-negative (Aβ−) based on ^18^F-florbetapir PET imaging findings*.* Unadjusted* P* values are presented, and those surviving multiple comparisons (Bonferroni’s correction, *P*_c_ < 0.05) are marked with an asterisk (*)

^a^Independent Student’s *t*-test. ^b^Chi-square test. ^c^Mann–Whitney *U* test. ^d^Generalized linear model adjusted for age and sex. ^e^One patient had missing data. ^f^Z-score transformed

*APOE* Apolipoprotein E, *PET* Positron emission tomography, *CDR* Clinical Dementia Rating, *MMSE* Mini-Mental State Examination, *MOCA* Montreal Cognitive Assessment, *FAQ* Functional Activities Questionnaire, *A/T/N* Amyloid/Tau/Neurodegeneration, *SUVR* Standardized uptake value ratio, *MTL* Medial temporal lobe, *NEO-T* Temporal neocortex, *metaROI* Meta-analytically derived region of interest, *t-tau* Total tau, *NfL* Neurofilament light chain

### Reciprocal associations between plasma and PET imaging ATN biomarkers

**Fig. 1:**
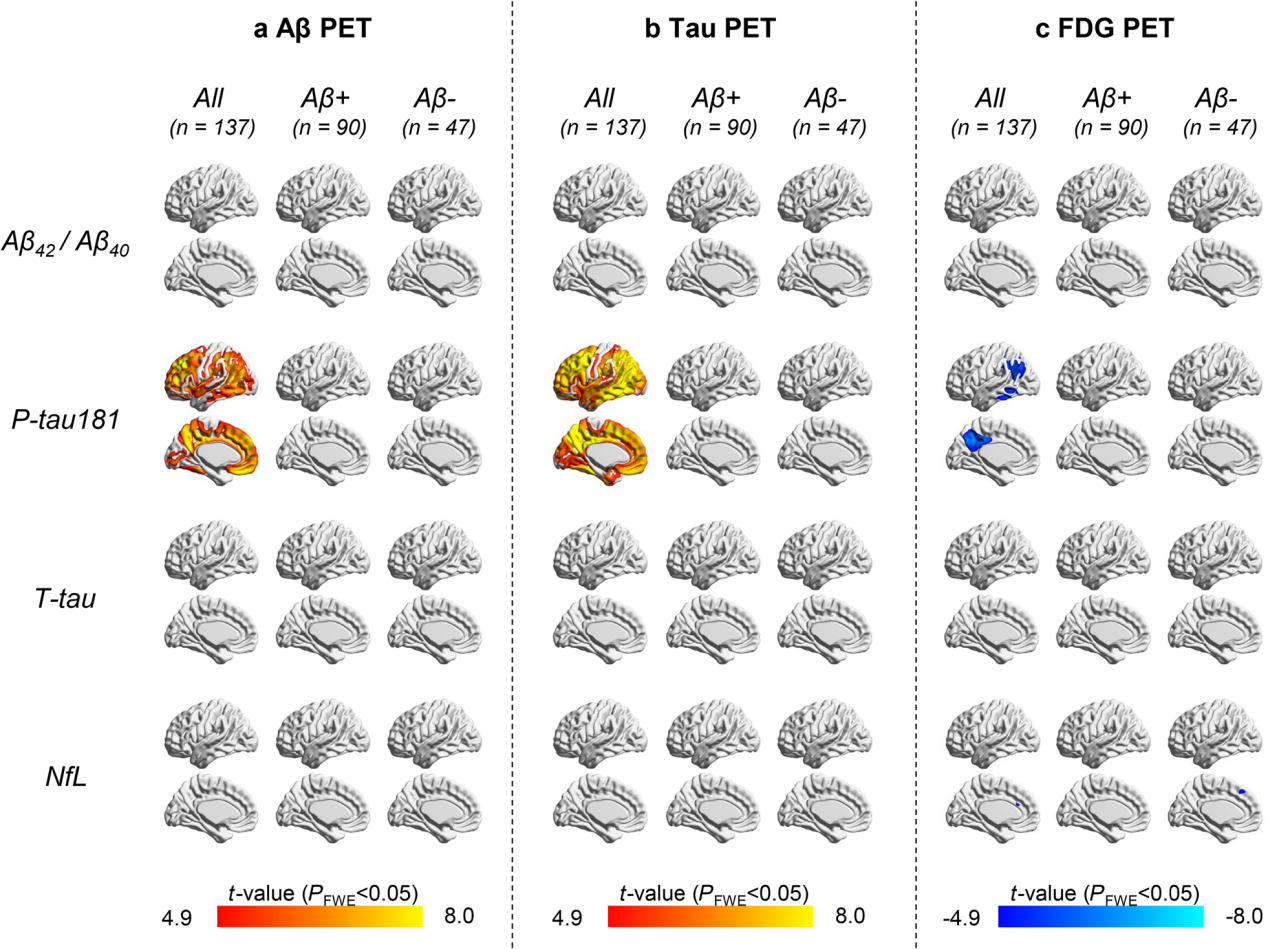
Reciprocal associations between plasma and PET imaging ATN biomarkers: voxel-wise analysis. Voxel-wise regression analysis of standardized uptake value ratios from ^18^F-florbetapir PET for A (**a**), ^18^F-Florzolotau PET for T (**b**), and ^18^F-FDG PET for N (**c**) in relation to plasma ATN biomarkers (Aβ_42_/Aβ_40_ ratio, p-tau181, t-tau, NfL) adjusted for age, sex, and the interval from PET imaging to blood collection; calculations were performed in the entire cohort, as well as in Aβ+ and Aβ− subjects. The statistical threshold was set at a family wise error (FWE)-corrected *P* value < 0.05. The positive correlations are displayed in orange-red color scale. The negative correlations are displayed in cyan-blue color scale. *PET* Positron emission tomography, *t*-tau, total tau, *NfL* Neurofilament light chain, *FWE* Family-wise error

**Fig. 2:**
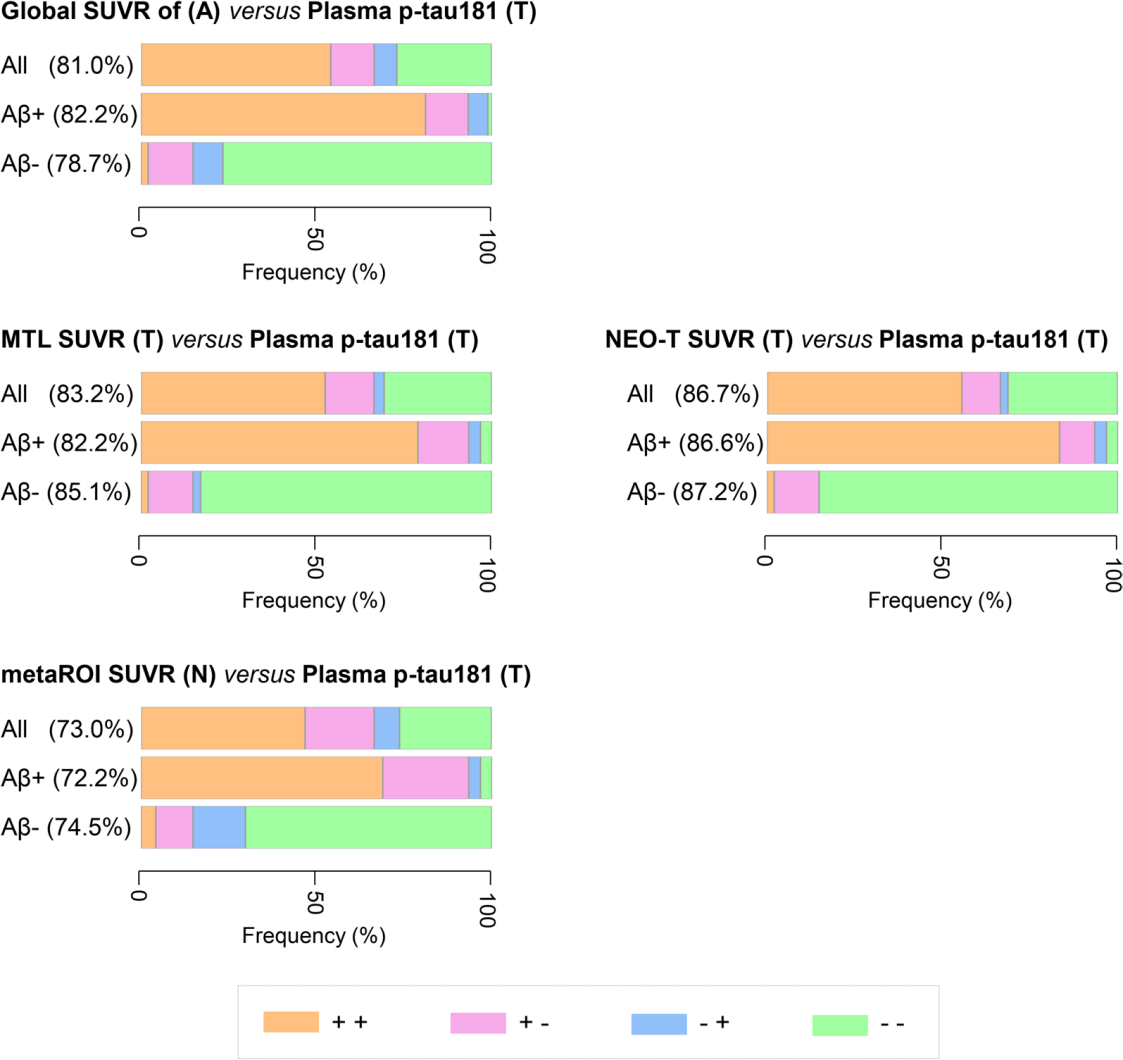
Agreement between plasma p-tau181 levels and PET imaging ATN biomarkers for predicting the Aβ status. The concordance rates are based on the established thresholds for the biomarkers. The sums of negative and positive concordance rates are presented. Color bars summarize the concordance rates between plasma p-tau181 level and different PET imaging ATN biomarkers in the entire cohort (upper row) as well as Aβ+ subjects (intermediate row) and Aβ− subjects (lower row). Negative (− −) and positive (+ +) agreement are denoted in green and orange, respectively. Disagreement is reported in magenta (+ −; positive SUVR value on PET and negative p-tau181 level) or in blue (− + ; negative SUVR value on PET and positive p-tau181 level). A/T/N, Amyloid/Tau/Neurodegeneration, *SUVR* Standardized uptake value ratio, *MTL* Medial temporal lobe, *NEO-T* Temporal neocortex; metaROI, meta-analytically derived region of interest; p-tau181, tau phosphorylated at threonine 181

The reciprocal associations between plasma and PET imaging ATN biomarkers analyzed on a voxel-wise level (*P*_FWE_ < 0.05) are shown in Fig. 1. In the entire cohort, the plasma p-tau181 level showed positive correlations with (1) Aβ PET SUVR value throughout the entire cortex – with only exception in the MTL as well as the precentral and postcentral gyrus (Fig. 1a) and (2) tau PET SUVR value throughout the entire cortex, with exception in the precentral and postcentral gyrus (Fig. 1b). Therefore, the findings of Aβ and tau PET imaging in the MTL differed significantly. Besides, the strength of the associations was lower for Aβ PET compared with tau PET imaging. On analyzing the ^18^F-FDG PET data, the plasma p-tau181 level was negatively correlated with SUVR values measured in the angular gyrus, precuneus, inferior parietal gyrus, as well as middle and posterior cingulate gyrus (Fig. 1c). No other significant associations between plasma and PET imaging ATN biomarkers were observed in the entire study cohort. In addition, all correlation analyses yielded negative results when Aβ+ and Aβ− patients were separately considered (Fig. 1). On a region-level basis (Table 2), the results were largely similar to those observed at the voxel-level. Consistent findings were seen when further adjusted for education and *APOE* ε4 (Additional file 1: Fig. S2, Table S1).

**Table 2:** Region-level reciprocal associations between plasma and PET imaging ATN biomarkers

	A Aβ_42_/Aβ_40_ ratio		T p-tau181, pg/ml		N t-tau, pg/ml		N NfL, pg/ml	
	*r*	*P*	*r*	*P*	*r*	*P*	*r*	*P*
*Entire cohort (n* = *137)*								
A: Global SUVR	− 0.044	0.617	0.528	< 0.001***	0.039	0.653	0.036	0.679
T: MTL SUVR	− 0.018	0.836	0.613	< 0.001***	0.078	0.373	0.172	**0.046**
T: NEO-T SUVR	− 0.035	0.686	0.623	< 0.001***	0.081	0.350	0.204	**0.018**
N: metaROI SUVR	0.026	0.767	− 0.491	< 0.001***	− 0.013	0.885	− 0.189	**0.029**
*Aβ+ (n* = *90)*								
A: Global SUVR	0.070	0.520	0.143	0.186	− 0.046	0.669	− 0.044	0.683
T: MTL SUVR	0.122	0.259	0.254	**0.018**	− 0.047	0.665	0.063	0.563
T: NEO-T SUVR	0.123	0.257	0.274	**0.010**	− 0.043	0.693	0.142	0.188
N: metaROI SUVR	− 0.146	0.178	− 0.217	**0.043**	0.171	0.112	− 0.042	0.700
*Aβ− (n* = *47)*								
A: Global SUVR	0.187	0.223	0.242	0.113	− 0.094	0.545	− 0.260	0.089
T: MTL SUVR	0.324	**0.032**	0.362	**0.016**	− 0.108	0.484	0.090	0.561
T: NEO-T SUVR	0.220	0.151	0.380	**0.011**	− 0.102	0.509	0.062	0.691
N: metaROI SUVR	− 0.013	0.931	-0.033	0.831	0.032	0.838	− 0.239	0.118

Partial correction analysis adjusted for age, sex, and the interval from PET imaging to blood collection was undertaken to assess the reciprocal associations between plasma and PET imaging ATN biomarkers. The reported *P* values are unadjusted. Significant *P* values (*P* < 0.05) are marked in bold, those surviving multiple comparisons (Bonferroni’s correction) are marked with asterisks (***, *P*_c_ < 0.001)

*PET* Positron emission tomography, *A/T/N* Amyloid/Tau/Neurodegeneration, *SUVR* Standardized uptake value ratio, *MTL* Medial temporal lobe, *NEO-T* Temporal neocortex, *metaROI* Meta-analytically derived region of interest, *t-tau* Total tau, *NfL* Neurofilament light chain

### Plasma and PET imaging ATN biomarkers in relation to the Aβ status

Compared with Aβ− participants, the Aβ+ participants consistently showed an increased pathological burden as reflected by higher PET SUVR values for AT biomarkers and lower PET SUVR value for N biomarker (*P*_c_ < 0.001; Table 1). Similar findings were observed for plasma biomarkers, with significantly higher p-tau181 level (*P*_c_ < 0.001) and a trend towards lower Aβ_42_/Aβ_40_ ratio (*P* = 0.054) and higher NfL level (*P* = 0.064) in the Aβ+ participants. However, plasma t-tau concentrations showed no intergroup difference. When further adjusted for education and *APOE* ε4 status, the differences in all PET biomarkers and plasma p-tau remained between Aβ+ and Aβ− subjects (*P*_c_ < 0.001) and there was still no difference in plasma t-tau (*P* = 0.308); however, the difference in plasma NfL reached the level of statistical significance (*P*_c_ < 0.039).

On analyzing the areas under the ROC curve (AUC) for distinguishing between Aβ+ and Aβ− subjects (Table 3), we found excellent diagnostic performances of the following biomarkers: global SUVR value for A (AUC = 0.93), MTL SUVR value for T (AUC = 0.94), NEO-T SUVR value for T (AUC = 0.95), and plasma p-tau181 level for T (AUC = 0.93). The accuracy of the metaROI SUVR value for N was less remarkable (AUC = 0.83), whereas plasma biomarkers for A and N lacked discriminatory ability (AUC ≤ 0.65). We next examined the agreement between plasma and PET imaging ATN biomarkers that were found to distinguish between Aβ+ and Aβ− patients. As expected (Fig. 2), plasma p-tau181 level for T and PET SUVR value for T showed a fairly high agreement (plasma p-tau181 level *versus* MTL SUVR value for T: Cohen’s kappa = 0.65; plasma p-tau181 level *versus* NEO-T SUVR value for T: Cohen’s kappa = 0.72). In addition, plasma p-tau181 level for T showed moderate agreement with both PET SUVR value for A (Cohen’s kappa = 0.59) and PET SUVR value for N (Cohen’s kappa = 0.45). Additional file 1: Fig. S3 shows a pairwise analysis of the observed agreement between different PET imaging ATN biomarkers – which ranged from substantial (A and T: Cohen’s kappa = 0.61 [global SUVR value for A *versus* MTL SUVR value for T], 0.70 [global SUVR value for A *versus* NEO-T SUVR value for T]) to moderate (T and N: Cohen’s kappa = 0.48 [NEO-T SUVR value for T *versus* metaROI SUVR value for N], 0.50 [MTL SUVR value for T *versus* metaROI SUVR value for N]) and fair (A and N: Cohen’s kappa = 0.33).

**Table 3:** Performance of plasma and PET imaging ATN biomarkers for predicting the Aβ status

	AUC (95% CI)	*P* value	Sensitivity (%)	Specificity (%)	Optimal cutoff
*PET ATN biomarkers*					
A: Global SUVR	0.93 (0.88–0.97)	< 0.001***	86.7	89.4	1.25
T: MTL SUVR	0.94 (0.82–0.96)	< 0.001***	82.2	95.7	1.30
T: NEO-T SUVR	0.95 (0.91–0.99)	< 0.001***	86.7	97.9	1.23
N: metaROI SUVR	0.83 (0.76–0.90)	< 0.001***	80.9	72.2	1.37
*Plasma ATN biomarkers*					
A: Aβ_42_/Aβ_40_ ratio	0.63 (0.54–0.73)	**0.011**	80.9	45.6	0.04
T: P-tau181, pg/ml	0.93 (0.88–0.98)	< 0.001***	93.3	85.1	3.23
N: T-tau, pg/ml	0.56 (0.46–0.66)	0.265	17.8	97.9	5.76
N: NfL, pg/ml	0.65 (0.54–0.75)	0.005*	81.1	51.1	13.63

Each participant was classified as either Aβ-positive (Aβ+) or Aβ-negative (Aβ−) based on ^18^F-florbetapir PET imaging findings. Each plasma and PET imaging ATN biomarker was examined by receiver operating characteristic (ROC) curve analysis in relation to its ability to predict the Aβ status. The optimal cutoff for each biomarker was selected as the point that maximized the Youden’s index according to the ROC curve analysis; the corresponding sensitivity and specificity were subsequently calculated. Unadjusted* P* values are presented. Significant* P* values (*P* < 0.05) are marked in bold, and those surviving multiple comparisons (Bonferroni’s correction) are marked with asterisks (***, *P*_c_ < 0.001; *,* P*_c_ < 0.05)

*PET* Positron emission tomography, *A/T/N* Amyloid/Tau/Neurodegeneration, *SUVR* Standardized uptake value ratio, *MTL* Medial temporal lobe, *NEO-T* Temporal neocortex, *metaROI *meta-analytically derived region of interest, *p-tau181* Tau phosphorylated at threonine 181, *t-tau* Total tau, *NfL* Neurofilament light chain, *AUC* Area under the curve, *CI* Confidence interval

### Plasma and PET imaging ATN biomarkers in relation to the severity of cognitive impairment

We next analyzed plasma and PET imaging ATN biomarkers in relation to the severity of cognitive impairment; to this aim, the study participants were categorized in different CDR categories.

**Fig. 3:**
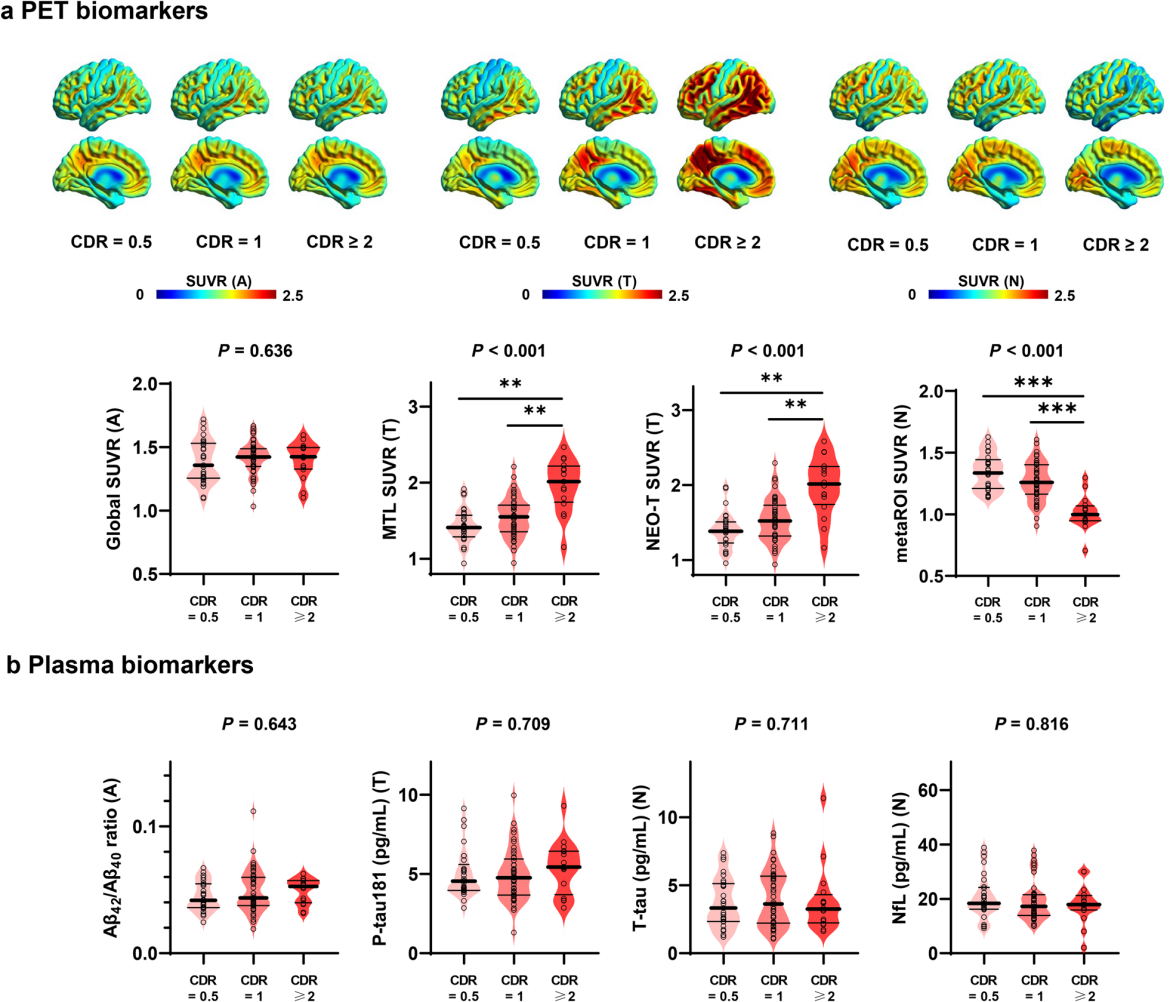
Plasma and PET imaging ATN biomarkers in relation to the severity of cognitive impairment in Aβ+ subjects. Average SUVR maps for PET imaging A (left), T (middle), and N (right) biomarkers in relation to different CDR categories (**a;** upper row). Generalized linear models after adjustment for age and sex were applied to analyze the values of PET (**a**; lower row) and plasma (**b**) ATN biomarkers in relation to the severity of cognitive impairment. Unadjusted *P* values are presented for differences between the three CDR categories, whereas those that remained significant after correcting for multiple comparisons (Bonferroni’s correction) are marked with asterisks (***, *P*_c_ < 0.001; **, *P*_c_ < 0.01). The thick solid line, the thin solid lines, and the dots denote the median, the 25th and 75th percentiles, and individual values, respectively. *CDR* Clinical Dementia Rating, *PET* Positron emission tomography, *A/T/N* Amyloid/Tau/Neurodegeneration, *SUVR* Standardized uptake value ratio, *MTL* Medial temporal lobe, *NEO-T* Temporal neocortex, *metaROI* Meta-analytically derived region of interest, *p*-tau181 tau phosphorylated at threonine 181, *t-tau* total tau, *NfL* Neurofilament light chain

The Aβ+ subjects were divided into three categories (CDR = 0.5,* n* = 29; CDR = 1, *n* = 45; CDR ≥ 2, *n* = 16). Group-average SUVR maps for each CDR category are presented in Fig. 3a. MTL SUVR value for T, NEO-T SUVR value for T and metaROI SUVR value for N showed that the tau burden increased and the glucose metabolism decreased in a stepwise fashion with increased CDR. Neither global SUVR value for A (Fig. 3a) nor plasma ATN biomarkers (Fig. 3b) showed such associations. On analyzing Aβ− subjects, we found that the CDR categories did not show significant associations with either plasma or PET imaging ATN biomarkers (Additional file 1: Fig. S4), with the only exceptions being metaROI SUVR value and plasma NfL level (both for N). Specifically, glucose hypometabolism showed a trend towards a higher frequency in patients with more severe cognitive impairment, although the difference did not persist after correcting for multiple comparisons. Plasma NfL level was significantly higher in patients with a CDR ≥ 1 than in those with a CDR = 0.5 (*P*_c_ < 0.01). The results from the entire cohort are shown in Additional file 1: Fig. S5. Similar results were obtained after corrections for education and *APOE* ε4 status (Additional file 1: Tables S2-S4).

### Plasma and PET imaging ATN biomarkers in relation to neuropsychological tests

**Fig. 4:**
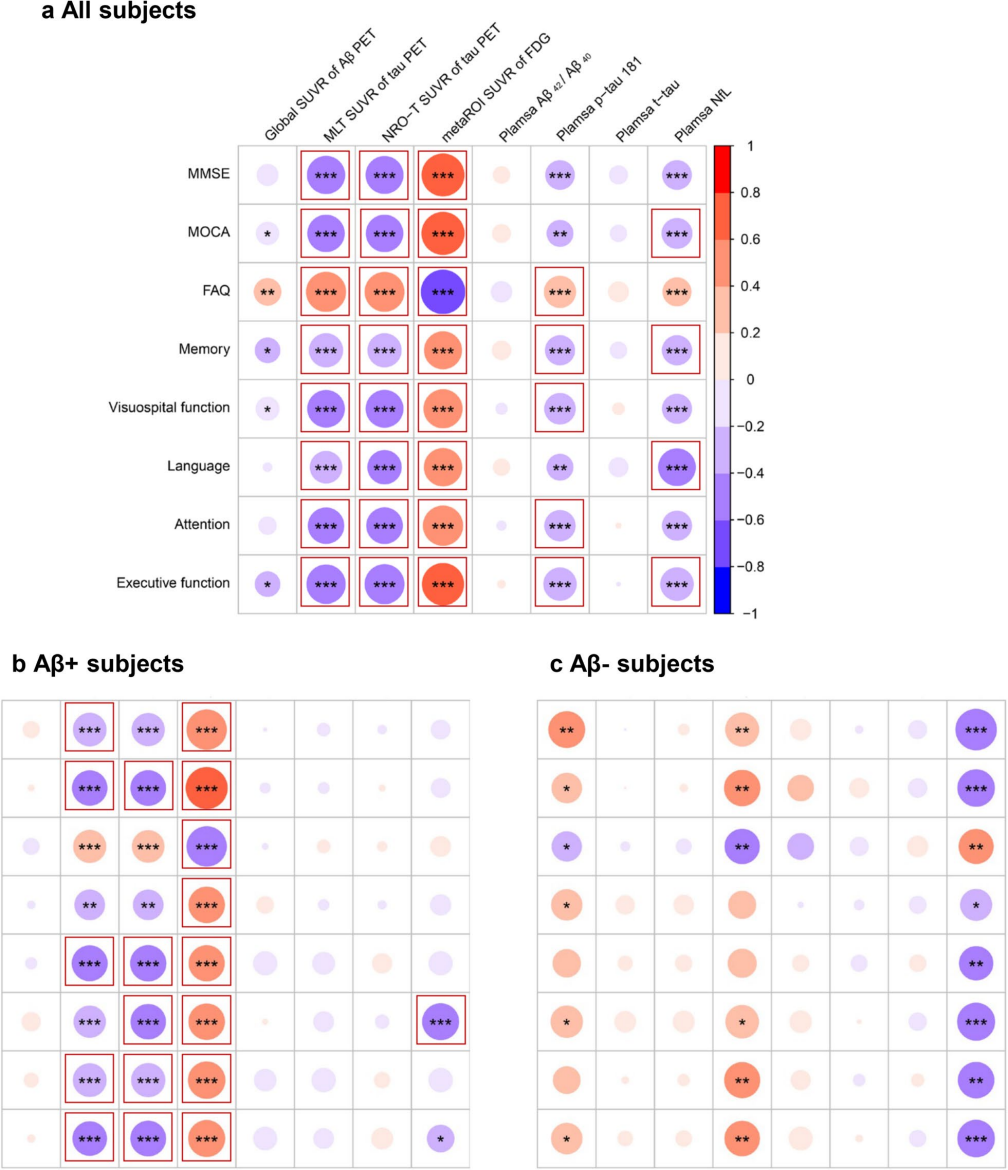
Associations of plasma and PET imaging ATN biomarkers with neuropsychological tests. Partial correction analysis after adjustment for age and sex was applied to evaluate the associations between plasma and PET imaging ATN biomarkers and the results of neuropsychological tests in the entire cohort (**a**) as well as in Aβ+ (**b**) and Aβ− (**c**) subjects. Unadjusted *P* values are presented with asterisks (***, *P* < 0.001; **, *P* < 0.01; *, *P* < 0.05) whereas those that remained significant after correcting for multiple comparisons (Bonferroni’s correction,* P*_c_ < 0.05) are marked with solid frames. The results of neuropsychological testing on different cognitive domains were transformed to Z-scores. The color bars denote partial correlation coefficients (*r*). *MMSE* Mini-Mental State Examination, *MOCA* Montreal Cognitive Assessment, *FAQ* Functional Activities Questionnaire, *PET* Positron emission tomography, *SUVR* Standardized uptake value ratio, *MTL* Medial temporal lobe; *NEO-T* Temporal neocortex, *metaROI* Meta-analytically derived region of interest, *t-tau* total tau, *NfL* Neurofilament light chain

On analyzing the entire study cohort, we found consistent associations between the results of neuropsychological tests and MTL SUVR value for T, NEO-T SUVR value for T, as well as metaROI SUVR value for N (Fig. 4a) – with statistical significance remaining after correcting for multiple comparisons. Similar significant associations were observed between plasma p-tau181 level for T and the results of the FAQ, as well as memory, visuospatial function, attention, and executive functioning. The plasma NfL level for N was significantly associated with the results of MOCA, as well as memory, language, and executive functioning. The results in the Aβ+ group (Fig. 4b) were generally consistent with those obtained in the entire cohort. However, no significant associations were observed when ATN biomarkers were analyzed in relation to neuropsychological tests in Aβ− subjects (Fig. 4c). Further corrections for education and *APOE* ε4 did not change the findings (Additional file 1: Fig. S6). The only exception was that in Aβ− subjects, the nonsignificant associations between plasma NfL level and scores of neuropsychological tests reached the significance after further adjustment for education and *APOE* ε4.

## Discussion

The present study has three main findings. First, plasma p-tau181 level was found to be significantly associated with PET imaging ATN biomarkers in the entire study cohort, although this association did not persist when Aβ+ and Aβ− subjects were analyzed separately. Second, we identified four biomarkers (global SUVR value for A, MTL SUVR value for T, NEO-T SUVR value for T, and plasma p-tau181 level for T) with similar and good performance in distinguishing between Aβ+ and Aβ− subjects. Third, we found that an increasing tau burden (as reflected by higher MTL and NEO-T SUVR values on ^18^F-Florzolotau PET) and a decreasing glucose metabolism (as reflected by lower metaROI SUVR value on ^18^F-FDG PET) were significantly associated with the severity of cognitive impairment in Aβ+ subjects. Glucose hypometabolism, along with elevated plasma NfL level, was also related to more severe cognitive impairment in Aβ− subjects. Taken together, while both ^18^F-Florzolotau tau PET and plasma p-tau181 are interchangeable markers for ^18^F-florbetapir amyloid PET on detecting the presence of amyloid pathology in unselected patients with cognitive complaints, plasma p-tau181 is preferred in screening considering the cost-effectiveness. Further, our study provides scoping information about the potential usefulness of ^18^F-Florzolotau PET and ^18^F-FDG PET as markers of clinical severity, and none of the plasma biomarkers included in the current study could be used interchangeably in this regard. Notably, since all participants had cognitive complaints and were recruited from a real-life memory clinic, the current findings may only apply to the symptomatic population.

In addition to the established imaging and CSF markers (Aβ PET imaging and CSF Aβ_42_/Aβ_40_ ratio) [40], plasma-based biomarkers have frequently been investigated for their ability to identify subjects with amyloid pathology – with most studies focusing on the plasma Aβ_42_/Aβ_40_ ratio. However, reliable studies have shown that the Aβ_42_/Aβ_40_ ratio in plasma generally underperforms the established CSF and imaging A biomarkers [41] and is prone to significant analytical variation [42]. By relying on the visual interpretation of ^18^F-florbetapir PET images to achieve a dichotomous classification of the Aβ status, our current findings further support the view that the plasma Aβ_42_/Aβ_40_ ratio has a limited value as an A biomarker [43]. Additionally, we found that neither plasma nor PET imaging A biomarkers were significantly associated with the severity of cognitive impairment. This finding is consistent with previous observations showing that cerebral Aβ accumulation reaches a plateau during the prodromal stage [44] and that the plasma amyloid biomarker profile does not correlate with cognitive function across the clinical spectrum of AD [45].

The agreement between PET, CSF, and plasma T biomarkers varies widely from 66% to 95% [46] and can be influenced by differences in assays and laboratory procedures [47]. The association of plasma p-tau181 (T) with CSF p-tau181 (T) as well as ^18^F-flortaucipir PET (T) and ^18^F-flutemetamol PET (A) has been previously reported for Aβ+ subjects and for an unselected sample comprising both Aβ+ and Aβ− individuals – but not for Aβ− subjects analyzed separately [11]. These findings are also consistent with an analysis by Thijssen et al*.* who reported significant associations of ^18^F-flortaucipir PET (T) with both plasma p-tau181 (T) and p-tau217 (T) [48]. In our study, associations of plasma p-tau181 (T) with ^18^F-Florzolotau PET (T), ^18^F-florbetapir PET (A), and ^18^F-FDG PET (N) were examined. However, they reached the threshold for statistical significance only in the entire study cohort. On analyzing Aβ+ individuals separately, our results were not consistent with previous studies possibly because we included only subjects with MCI and dementia due to AD but not those in the preclinical stage, as other authors did [11, 48].

Our data also showed that the plasma p-tau181 level (T) was more closely associated with ^18^F-Florzolotau PET (T) than ^18^F-florbetapir PET (A), although Thijssen et al*.* found that the plasma p-tau biomarkers were mainly related to PET imaging A biomarkers (^18^F-AZD4694, ^18^F-florbetapir) than T biomarkers (^18^F-MK6240, ^18^F-flortaucipir) [48]. This discrepancy might be because that the present study used a different tau PET tracer (^18^F-Florzolotau) and did not include subjects in the preclinical stage. It is worth noting that ^18^F-Florzolotau has shown favorable affinity to all types of tau aggregates [21] and is able to detect tau deposition in vivo in the brains of patients with different tauopathies (i.e., three- and four-repeat (3R/4R) tau in AD [49–51], 4R-tau in progressive supranuclear palsy [31, 52], 4R and 3R/4R-tau in frontotemporal lobar degeneration with tauopathy caused by microtubule-associated protein tau mutations [53]) while ^18^F-MK6240 and ^18^F-flortaucipir have relatively low affinity for non-AD tauopathies [54–56].

Plasma levels of p-tau biomarkers have been reported to be strongly correlated with both CSF and PET imaging T biomarkers [11, 14, 15, 46, 48, 57]. In line with these findings, the present study found similar patterns for plasma p-tau181 concentrations and PET imaging results with a second-generation tau tracer (^18^F-Florzolotau). Interestingly, these markers also appeared to have an excellent discriminatory ability to distinguish between Aβ+ and Aβ− individuals. Research investigating plasma (p-tau181, p-tau217, and p-tau231) and PET imaging (^18^F-RO948, ^18^F-MK-6240, and ^18^F-flortaucipir) T biomarkers has generally detected an increasing tau burden from the preclinical stage to clinically overt dementia. Aside from evidence that plasma p-tau levels tend to increase in a less pronounced fashion in symptomatic patients [11, 15, 45, 58–61], the correlations of plasma T biomarkers with the results of neuropsychological testing are generally moderate [17, 45, 48]. The pathophysiological cascade of Aβ- and tau-related processes is not constant during disease progression, that is, as opposed to early in the disease, in the advanced stages such as AD dementia when Aβ fibrils and soluble p-tau levels have stabilized, cognitive decline is associated with the accumulation rate of insoluble tau aggregates [62]. Our data add to previous evidence by demonstrating that only SUVR values on ^18^F-Florzolotau PET imaging – and not plasma T biomarker – increased in a stepwise fashion with the increasing severity of cognitive impairment. This result suggests that plasma and PET imaging T biomarkers may convey information that is at least in part not overlapping, with plasma p-tau181 concentrations being more closely related to Aβ pathology and tau PET imaging findings being mainly a reflection of the cognitive impairment severity [46]. However, this possibility requires confirmation given the non-linear increase in plasma p-tau181 concentrations during the course of AD [63]. Another prospective research with different T biomarkers from those of our study consistently indicated that the soluble tau as reflected by elevated plasma p-tau217 and the insoluble tau aggregates as reflected by elevated tau PET (^18^F-RO948 and ^18^F-flortaucipir) signals, are optimal predictors for longitudinal tau accumulation in the brains of patients with AD at preclinical and prodromal phases, respectively [18]. Future work in this area should also validate different plasma T biomarkers, which may have differential roles for identifying amyloid pathology [64]. Moreover, since comorbidities such as chronic kidney disease are reported to have a non-negligible impact on the interpretation of plasma p-tau181 and p-tau217 levels [16], further studies exploring their potential impact on tau PET biomarkers are warranted.

Given that neurodegeneration is the final consequence of various pre-existing pathological alterations, research has generally explored the association of N biomarkers with the severity of cognitive impairment and clinical trajectories over time [65]. However, ^18^F-FDG PET as an imaging N biomarker also shows diagnostic value among patients present to memory clinics with an uncertain diagnosis [66]. In line with prior studies [14, 67], metaROI SUVR value on ^18^F-FDG PET imaging was the only N biomarker capable of distinguishing between Aβ+ and Aβ− individuals, although it underperformed both A and T biomarkers. There is also evidence that, different from other N biomarkers, glucose hypometabolism on ^18^F-FDG PET may predict a steeper cognitive decline trajectory; therefore, the traditional classification of ^18^F-FDG PET imaging as an N biomarker has been put into question [68]. Interestingly, we found that ^18^F-FDG PET outperformed NfL – a plasma N biomarker – in reflecting the severity of cognitive impairment in Aβ+ individuals. However, the plasma NfL level was superior to ^18^F-FDG PET as a marker of disease severity in Aβ− individuals – a finding which calls for additional investigations.

While we are not aware of any other study that has provided a head-to-head comparison of plasma and PET imaging ATN biomarkers in relation to the presence of amyloid pathology and the severity of cognitive impairment across the AD spectrum, several design limitations should be acknowledged. Since this single-center investigation was cross-sectional, it is not possible to establish the causal nature or the directionality of the observed associations. We did not obtain longitudinal measures of cognitive impairment, which restricts the prognostic impact of our findings. Meanwhile, the sample size of the final study cohort was limited, and attention needs to be paid to potential sources of bias. We have only enrolled patients presented to a memory clinic and consequently we were unable to include subjects in the preclinical stage. Our findings may be most applicable and generalizable to those with MCI or dementia due to AD. Another limitation is the uneven distribution of amyloid pathology, resulting in more participants within the Aβ+ group. The uneven distributions of different severities of clinical cognitive impairment, a common drawback of the serial-enrollment design when the study sample size is limited, also requires attention. The present analysis did not include measurements of recently developed plasma T biomarkers (i.e., p-tau217, p-tau231), as well as of neuroinflammatory markers. Because the availability of ^18^F-Florzolotau PET imaging is still limited, our results are not conducive to establishing a definitive equivalence between this imaging modality and the combination of plasma p-tau181 and ^18^F-FDG PET. Besides, two atlases were used for PET imaging analysis, which may have attenuated some of the results although preceding data rendered such effects likely to be minimal [69]. Last but not least, we took the visual assessment of ^18^F-florbetapir PET imaging as a ground truth for Aβ status. As semi-quantitative binary cutoffs (i.e., a global SUVR greater than 1.1 indicates positive Aβ accumulation) [34, 70] have been recommended for ^18^F-florbetapir, it is necessary to further replicate our findings using semi-quantitative measurements as the ground truth.

## Conclusion

The results from the present study raise the possibility that ^18^F-florbetapir PET imaging (A), ^18^F-Florzolotau PET imaging (T), and plasma p-tau181 (T) can be considered as interchangeable biomarkers in the assessment of Aβ status in both MCI and dementia due to AD. The findings on ^18^F-Florzolotau PET (T) and ^18^F-FDG PET (N) could also serve as imaging markers for the severity of cognitive impairment.

## Supplementary Information

**Additional file 1 Fig. S1.** Study flowchart. **Fig. S2.** Reciprocal associations between plasma and PET imaging ATN biomarkers: voxel-wise analysis. **Fig. S3.** Agreement between different PET imaging ATN biomarkers for predicting the Aβ status. **Fig. S4.** Plasma and PET imaging ATN biomarkers in relation to the severity of cognitive impairment in Aβ− subjects. **Fig. S5.** Plasma and PET imaging ATN biomarkers in relation to the severity of cognitive impairment in the entire cohort. **Fig. S6.** Associations of plasma and PET imaging ATN biomarkers with neuropsychological tests. **Table S1.** Region-level reciprocal associations between plasma and PET imaging ATN biomarkers. **Table S2.** Plasma and PET imaging ATN biomarkers in relation to the severity of cognitive impairment in Aβ+ subjects. **Table S3.** Plasma and PET imaging ATN biomarkers in relation to the severity of cognitive impairment in Aβ− subjects. **Table S4.** Plasma and PET imaging ATN biomarkers in relation to the severity of cognitive impairment in the entire cohort.

## Data Availability

Fully anonymized data will be shared upon request from qualified investigators, subject to approval by the China Human Genetic Resources Administration Office. Data transfer will have to comply with the Regulations of the People’s Republic of China.

## Abbreviations

AAL3: Automated Anatomical Labelling Atlas three
Aβ: β-Amyloid
AD: Alzheimer’s disease
*APOE*: Apolipoprotein E
CDR: Clinical dementia rating
CSF: Cerebrospinal fluid
FAQ: Functional Assessment Questionnaire
FDG: Fluorodeoxyglucose
FWE: Family wise error
GLM: Generalized linear model
MCI: Mild cognitive impairment
metaROI: Meta-analytically derived region of interest
MMSE: Mini-Mental State Examination
MOCA: Montreal Cognitive Assessment
MRI: Magnetic resonance imaging
MTL: Medial temporal lobe
NEO-T: Temporal neocortex
NfL: Neurofilament light chain
PET: Positron emission tomography
p-tau: Phosphorylated tau
SCD: Subjective cognitive decline
SPM12: Statistical Parametric Mapping 12
SUVR: Standardized uptake value ratio
