# Supplementary material for: Head-to-head comparison of plasma and PET imaging ATN markers in subjects with cognitive complaints
Source: Transl Neurodegener. 2023 Jun 29;12:34. doi: 10.1186/s40035-023-00365-x (PMC10308642; doi:10.1186/s40035-023-00365-x)
Supplement: Supplementary file 1 — Additional file 1 Fig. S1. Study flowchart. Fig. S2. Reciprocal associations between plasma and PET imaging ATN biomarkers: voxel-wise analysis. Fig. S3. Agreement between different PET imaging ATN biomarkers for predicting the Aβ status. Fig. S4. Plasma and PET imaging ATN biomarkers in relation to the severity of cognitive impairment in Aβ− subjects. Fig. S5. Plasma and PET imaging ATN biomarkers in relation to the severity of cognitive impairment in the entire cohort. Fig. S6. Associations of plasma and PET imaging ATN biomarkers with neuropsychological tests. Table S1. Region-level reciprocal associations between plasma and PET imaging ATN biomarkers. Table S2. Plasma and PET imaging ATN biomarkers in relation to the severity of cognitive impairment in Aβ+ subjects. Table S3. Plasma and PET imaging ATN biomarkers in relation to the severity of cognitive impairment in Aβ− subjects. Table S4. Plasma and PET imaging ATN biomarkers in relation to the severity of cognitive impairment in the entire cohort. [file 40035_2023_365_MOESM1_ESM.docx]

**Additional file 1**


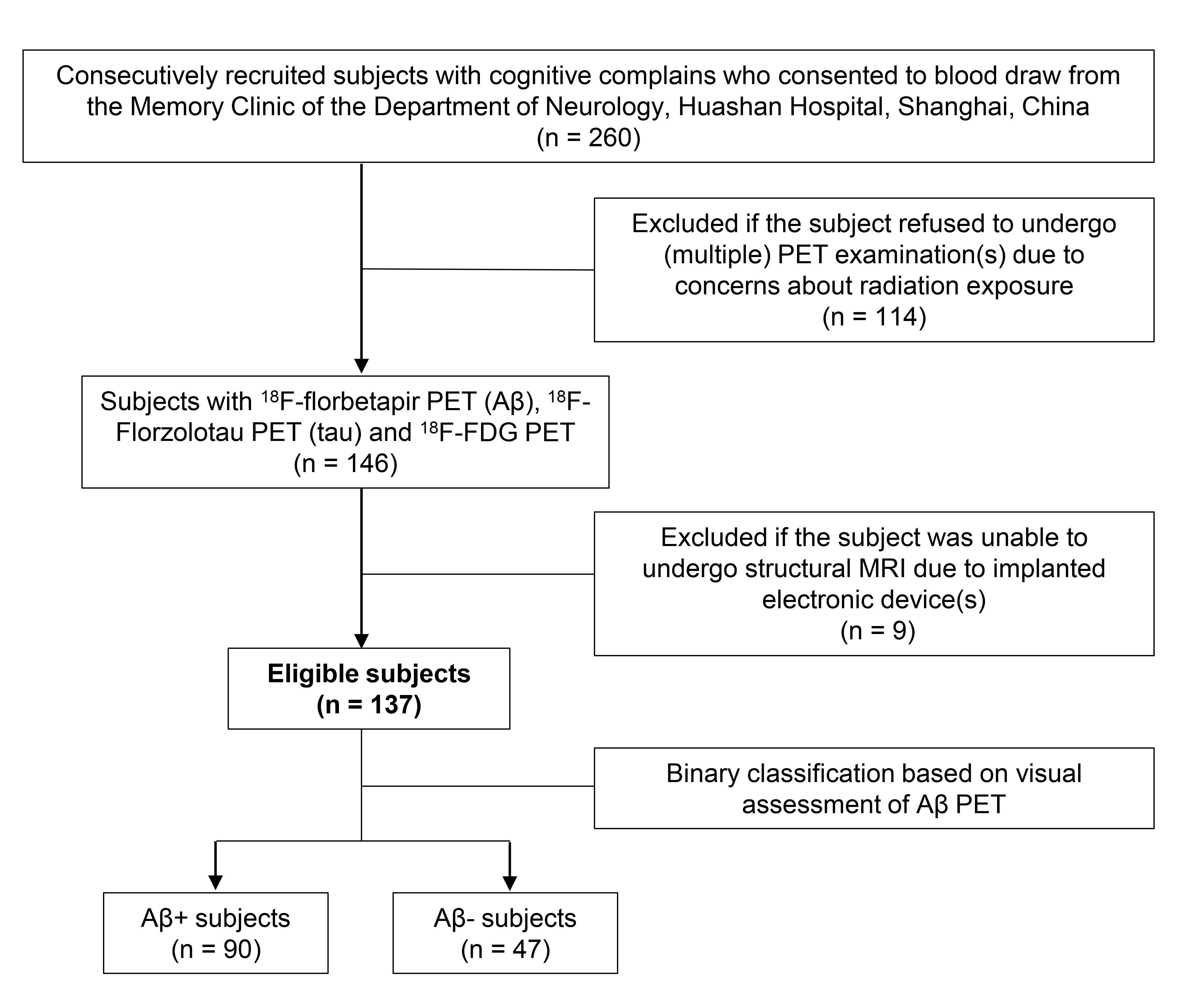


**Fig. S1 Study flowchart.** Each participant was classified as either Aβ-positive (Aβ+) or Aβ-negative (Aβ-) based on ^18^F-florbetapir PET imaging findings. Aβ+, β-amyloid positive; Aβ-, β-amyloid negative; PET, positron emission tomography; MRI, magnetic resonance imaging.

**Fig. S2** Reciprocal associations between plasma and PET imaging ATN biomarkers: voxel-wise analysis (covariates: age, sex, the interval from PET imaging to blood collection, education and *APOE* ε4). Voxel-wise regression analysis of standardized uptake value ratios from ^18^F-florbetapir PET for A **(a)**, ^18^F-Florzolotau PET for T **(b)**, and ^18^F-FDG PET for N **(c)** in relation to plasma ATN biomarkers (Aβ_42_/Aβ_40_ ratio, p-tau181, t-tau, NfL) adjusted for age, sex, the interval from PET imaging to blood collection, education and *APOE* ε4; calculations were performed in the entire cohort, as well as in Aβ+ and Aβ- subjects. The statistical threshold was set at a family wise error (FWE)-corrected *P* value < 0.05. The positive correlations, that is, the higher PET SUVR is, the higher plasma biomarker level is, are displayed in orange-red color scale. The negative correlations, that is, the lower PET SUVR is, the higher plasma biomarker level is, are displayed in cyan-blue color scale. Aβ+, β-amyloid positive; Aβ-, β-amyloid negative; *APOE*, apolipoprotein E; PET, positron emission tomography; Aβ, β-amyloid; p-tau181, tau phosphorylated at threonine 181; t-tau, total tau; NfL, neurofilament light chain; FWE, family-wise error.

**
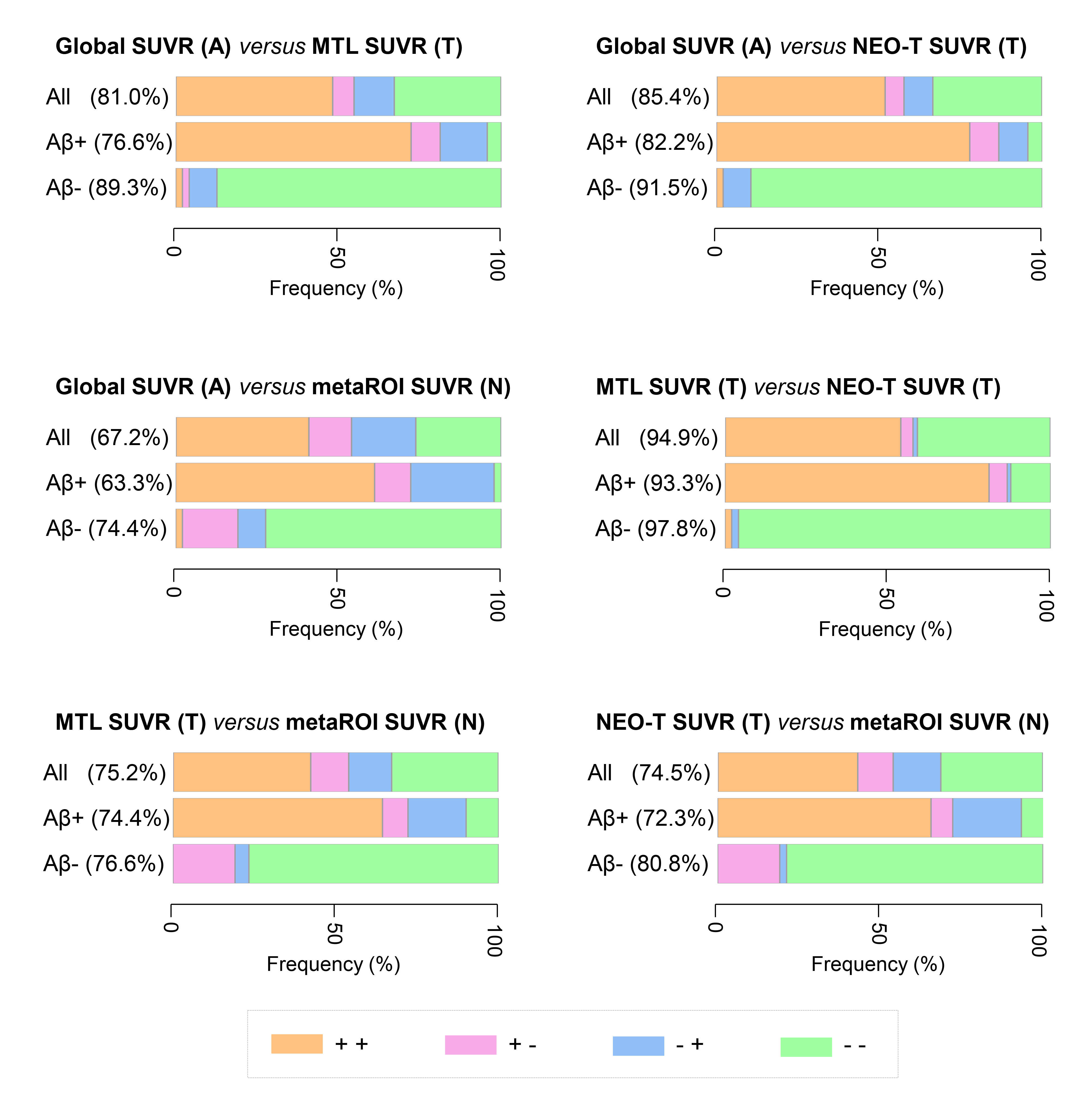
**

**Fig. S3 Agreement between different PET imaging ATN biomarkers for predicting the Aβ status.** Color bars summarize the concordance rates between different PET imaging ATN biomarkers in the entire cohort (upper row) as well as Aβ+ subjects (intermediate row) and Aβ- subjects (lower row), respectively. Negative (--) and positive (++) agreement are denoted in green and orange, respectively. Disagreement is reported in magenta (+-) or in blue (-+). The optimal cutoff for each biomarker was selected as the point that maximized the Youden’s index. The sums of negative and positive concordance rates are presented. A/T/N, Amyloid/Tau/Neurodegeneration; SUVR, standardized uptake value ratio; MTL, medial temporal lobe; NEO-T, temporal neocortex; metaROI, meta-analytically derived region of interest; Aβ+, β-amyloid positive; Aβ-, β-amyloid negative.


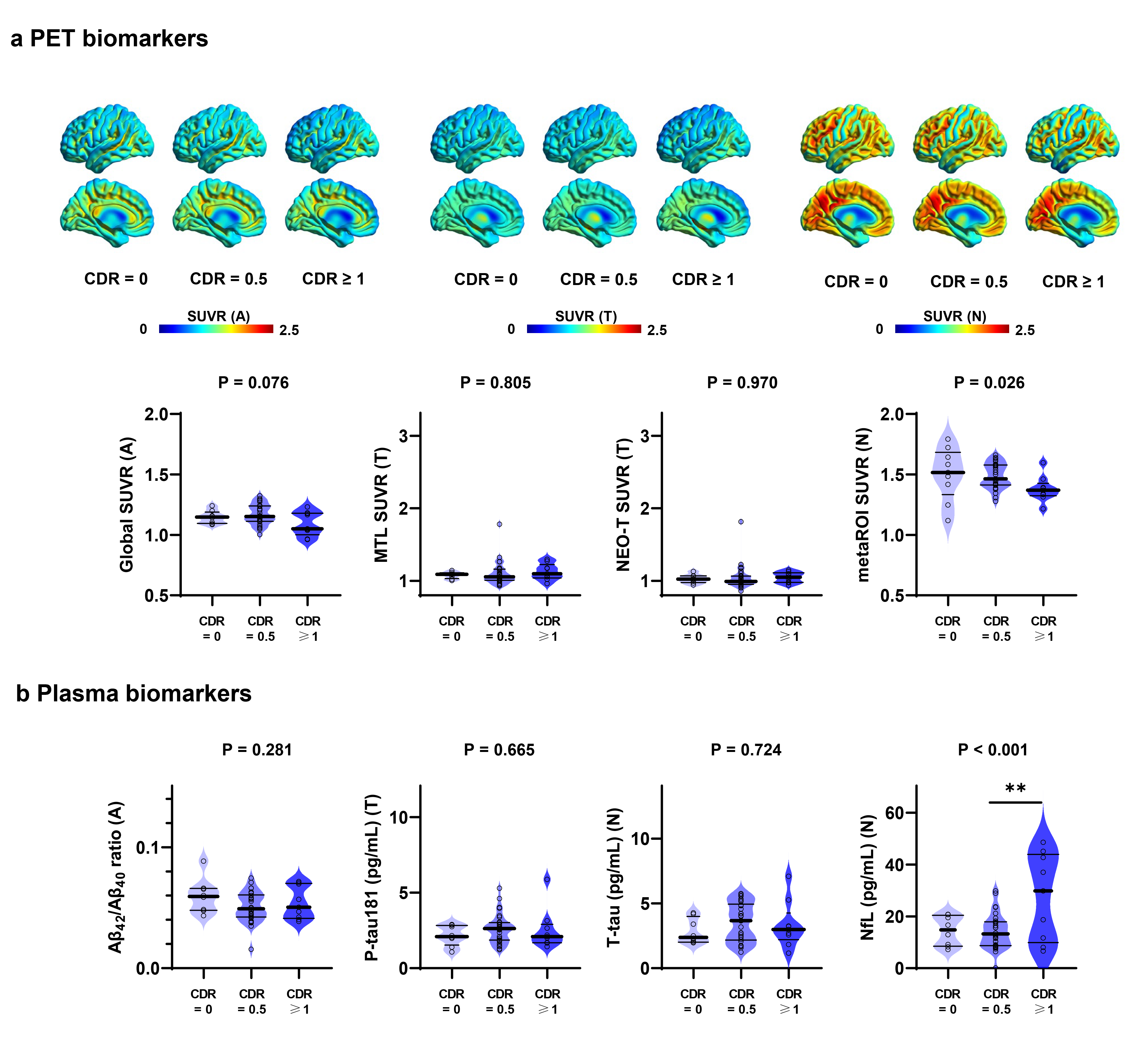


**Fig. S4. Plasma and PET imaging ATN biomarkers in relation to the severity of cognitive impairment in Aβ- subjects.** Average SUVR maps for PET imaging A (left), T (middle), and N (right) biomarkers in relation to different CDR categories **(a;** upper row**)**. Generalized linear models after adjustment for age and sex were applied to analyze the values of PET (**a**; lower row) and plasma **(b)** ATN biomarkers in relation to the severity of cognitive impairment. Unadjusted P values are presented for differences between the three CDR categories, whereas those that remained significant after correcting for multiple comparisons (Bonferroni’s correction) are marked with asterisks (**, *P_c_* < 0.01). The thick solid line, the thin solid lines, and the dots denote the median, the 25th and 75th percentiles, and individual values, respectively. CDR, Clinical Dementia Rating; PET, positron emission tomography; A/T/N, Amyloid/Tau/Neurodegeneration; SUVR, standardized uptake value ratio; SUVR, standardized uptake value ratio; MTL, medial temporal lobe; NEO-T, temporal neocortex; metaROI, meta-analytically derived region of interest; Aβ, β-amyloid; p-tau181, tau phosphorylated at threonine 181; t-tau, total tau; NfL, neurofilament light chain.


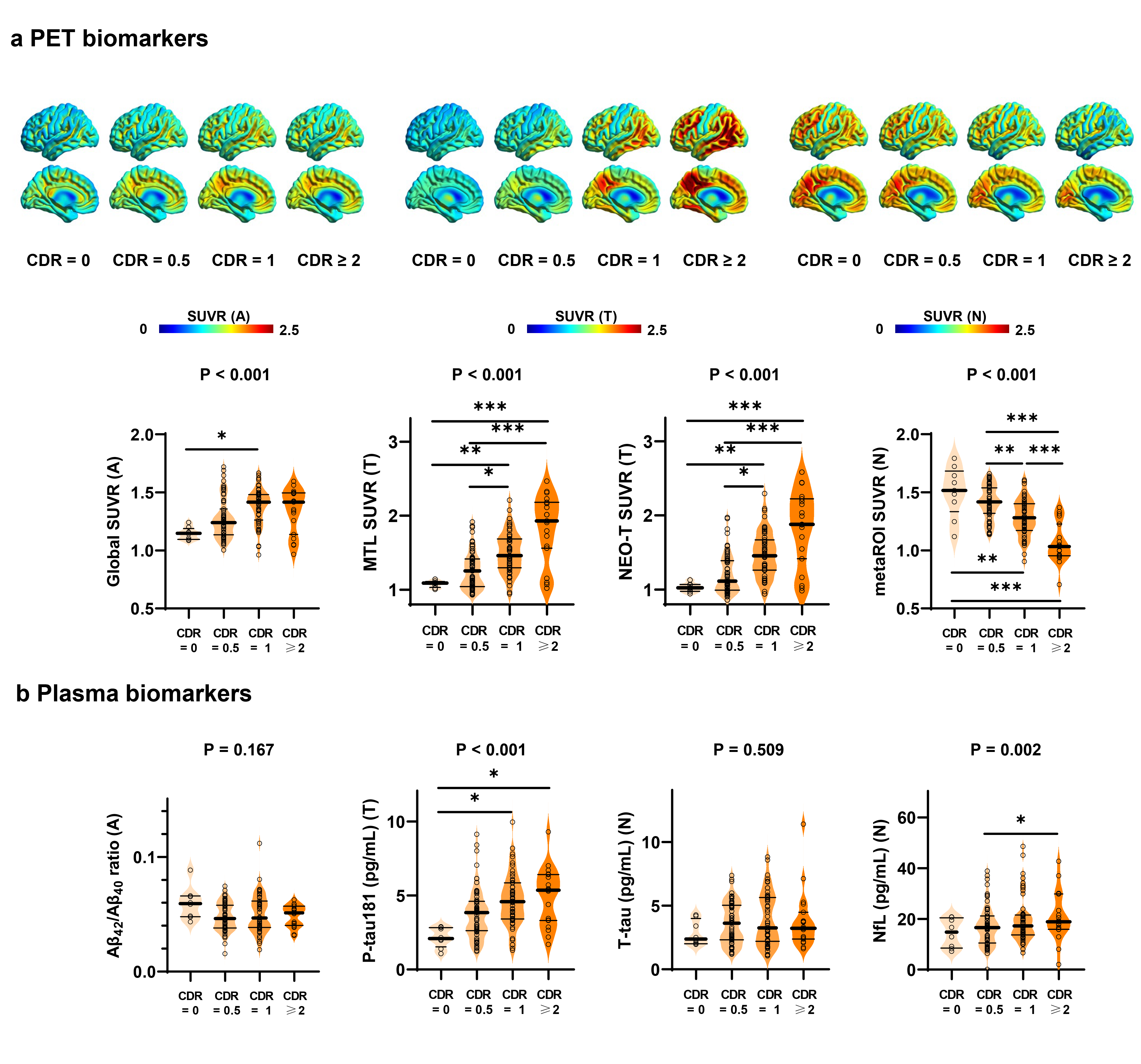


**Fig. S5. Plasma and PET imaging ATN biomarkers in relation to the severity of cognitive impairment in the entire cohort.** Average SUVR maps for PET imaging A (left), T (middle), and N (right) biomarkers in relation to different CDR categories **(a;** upper row**)**. The number (%) of amyloid-positive subjects in the four subgroups (CDR = 0, = 0.5, = 1, ≥ 2) was 8 (0%), 59 (49.2%), 51 (88.2%), and 19 (84.2%), respectively. Generalized linear models after adjustment for age and sex were applied to analyze the values of PET (**a**; lower row) and plasma **(b)** ATN biomarkers in relation to the severity of cognitive impairment. Unadjusted P values are presented for differences between the four CDR categories, whereas those that remained significant after correcting for multiple comparisons (Bonferroni’s correction) are marked with asterisks (***, *P_c_* < 0.001; **, *P_c_* < 0.01; *, *P_c_* < 0.05). The thick solid line, the thin solid lines, and the dots denote the median, the 25th and 75th percentiles, and individual values, respectively. CDR, Clinical Dementia Rating; PET, positron emission tomography; A/T/N, Amyloid/Tau/Neurodegeneration; SUVR, standardized uptake value ratio; SUVR, standardized uptake value ratio; MTL, medial temporal lobe; NEO-T, temporal neocortex; metaROI, meta-analytically derived region of interest; Aβ, β-amyloid; p-tau181, tau phosphorylated at threonine 181; t-tau, total tau; NfL, neurofilament light chain.


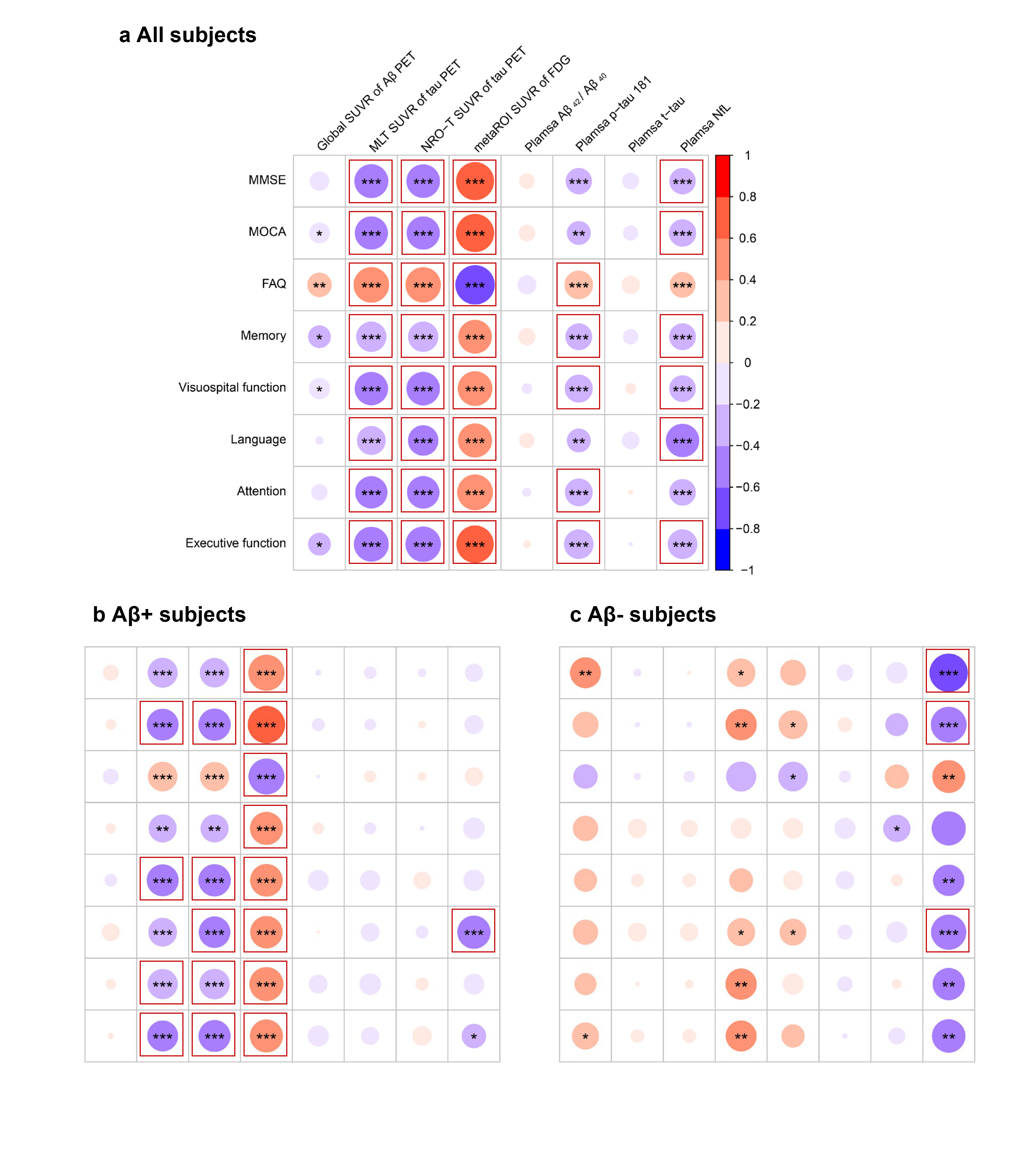


**Fig. S6.** Associations of plasma and PET imaging ATN biomarkers with neuropsychological tests (covariates: age, sex, education and *APOE* ε4).

Partial correction analysis after adjustment for age, sex, education and *APOE* ε4was applied to evaluate the associations between plasma and PET imaging ATN biomarkers and the results of neuropsychological tests in the entire cohort **(a)** as well as in Aβ+ **(b)** and Aβ- **(c)** subjects. Unadjusted P values are presented with asterisks (***, *P* < 0.001; **, *P* < 0.01; *, *P* < 0.05) whereas those that remained significant after correcting for multiple comparisons (Bonferroni’s correction, *P_c_* < 0.05) are marked with solid frames. The results of neuropsychological testing on different cognitive domains were transformed to Z-scores. The color bars denote partial correlation coefficients (r). *APOE*, apolipoprotein E; MMSE, Mini-Mental State Examination; MOCA, Montreal Cognitive Assessment; FAQ, Functional Activities Questionnaire; PET, positron emission tomography; SUVR, standardized uptake value ratio; MTL, medial temporal lobe; NEO-T, temporal neocortex; metaROI, meta-analytically derived region of interest; Aβ, β-amyloid; p-tau181, tau phosphorylated at threonine 181; t-tau, total tau; NfL, neurofilament light chain.

**Table S1. Region-level reciprocal associations between plasma and PET imaging ATN biomarkers (covariates: age, sex, the interval from PET imaging to blood collection, education and *APOE* ε4).**

|  | A  Aβ_42_/Aβ_40_ ratio | | T  P-tau181, pg/mL | | N  T-tau, pg/mL | | N  NfL, pg/mL | |
| --- | --- | --- | --- | --- | --- | --- | --- | --- |
|  | *r* | *P* | *r* | *P* | *r* | *P* | *r* | *P* |
| **Entire cohort (*n* = 136)** | | | | | | | | |
| A: Global SUVR | -0.016 | 0.858 | 0.497 | **<0.001***** | 0.031 | 0.724 | 0.075 | 0.395 |
| T: MTL SUVR | 0.001 | 0.991 | 0.596 | **<0.001***** | 0.074 | 0.401 | 0.208 | **0.017** |
| T: NEO-T SUVR | -0.018 | 0.837 | 0.607 | **<0.001***** | 0.077 | 0.381 | 0.238 | **0.006** |
| N: metaROI SUVR | 0.015 | 0.867 | -0.491 | **<0.001***** | -0.009 | 0.923 | -0.200 | **0.022** |
| **Aβ+ (*n* = 89)** |  |  |  |  |  |  |  |  |
| A: Global SUVR | 0.104 | 0.345 | 0.154 | 0.163 | -0.050 | 0.654 | -0.042 | 0.704 |
| T: MTL SUVR | 0.133 | 0.228 | 0.261 | **0.017** | -0.041 | 0.710 | 0.060 | 0.590 |
| T: NEO-T SUVR | 0.132 | 0.233 | 0.279 | **0.010** | -0.036 | 0.746 | 0.134 | 0.226 |
| N: metaROI SUVR | -0.163 | 0.137 | -0.218 | **0.046** | 0.170 | 0.122 | -0.006 | 0.959 |
| **Aβ- (*n* = 47)** |  |  |  |  |  |  |  |  |
| A: Global SUVR | 0.272 | 0.081 | 0.190 | 0.227 | -0.107 | 0.502 | -0.229 | 0.145 |
| T: MTL SUVR | 0.345 | **0.025** | 0.358 | **0.020** | -0.112 | 0.480 | 0.101 | 0.523 |
| T: NEO-T SUVR | 0.247 | 0.116 | 0.371 | **0.016** | -0.112 | 0.480 | 0.073 | 0.645 |
| N: metaROI SUVR | 0.024 | 0.879 | -0.066 | 0.677 | 0.006 | 0.968 | -0.239 | 0.127 |

Partial correction analysis adjusted for age, sex, the interval from PET imaging to blood collection, education and *APOE* ε4 was undertaken to assess the reciprocal associations between plasma and PET imaging ATN biomarkers. One participant from Aβ+ group did not have *APOE* ε4 information and was excluded. The reported P values are unadjusted. Significant P values (*P* < 0.05) are marked in bold, whereas an asterisk denotes those that retained significance after adjustment for multiple comparisons (Bonferroni’s correction) are marked with asterisks (***, *P_c_* < 0.001). Aβ+, β-amyloid positive; Aβ-, β-amyloid negative; *APOE*, apolipoprotein E; PET, positron emission tomography; A/T/N, Amyloid/Tau/Neurodegeneration; SUVR, standardized uptake value ratio; MTL, medial temporal lobe; NEO-T, temporal neocortex; metaROI, meta-analytically derived region of interest; Aβ, β-amyloid; p-tau181, tau phosphorylated at threonine 181; t-tau, total tau; NfL, neurofilament light chain.

**Table S2. Plasma and PET imaging ATN biomarkers in relation to the severity of cognitive impairment in Aβ+ subjects (covariates: age, sex, education and *APOE* ε4).**

| *P* value | Overall | CDR = 0.5 *versus* CDR = 1 | CDR = 0.5 *versus* CDR ≥ 2 | CDR = 1  *versus* CDR ≥ 2 |
| --- | --- | --- | --- | --- |
| **PET biomarkers** |  |  |  |  |
| A: Global SUVR | 0.690 | 0.447 | 0.952 | 0.593 |
| T: MTL SUVR | **<0.001** | 0.465 | **<0.001**** | **<0.001**** |
| T: NEO-T SUVR | **<0.001** | 0.429 | **<0.001*** | **<0.001*** |
| N: metaROI SUVR | **<0.001** | 0.164 | **<0.001***** | **<0.001***** |
| **Plasma biomarkers** |  |  |  |  |
| A: Aβ_42_/Aβ_40_ ratio | 0.579 | 0.297 | 0.600 | 0.824 |
| T: P-tau181, pg/mL | 0.698 | 0.427 | 0.863 | 0.667 |
| N: T-tau, pg/mL | 0.751 | 0.680 | 0.748 | 0.486 |
| N: NfL, pg/mL | 0.780 | 0.985 | 0.575 | 0.508 |

Generalized linear models after adjustment for age, sex, education and *APOE* ε4 were applied to analyze the values of PET and plasma ATN biomarkers in relation to the severity of cognitive impairment. Unadjusted P values are presented for differences between the three CDR categories, those that reach significance (P < 0.05) before multiple comparisons adjustment are marked in bold, those that remain significant after multiple comparisons corrections (Bonferroni’s correction) are marked with asterisks (***, *P_c_* < 0.001; **, *P_c_* < 0.01; *, *P_c_* < 0.05). *APOE*, apolipoprotein E; CDR, Clinical Dementia Rating; PET, positron emission tomography; A/T/N, Amyloid/Tau/Neurodegeneration; SUVR, standardized uptake value ratio; SUVR, standardized uptake value ratio; MTL, medial temporal lobe; NEO-T, temporal neocortex; metaROI, meta-analytically derived region of interest; Aβ, β-amyloid; p-tau181, tau phosphorylated at threonine 181; t-tau, total tau; NfL, neurofilament light chain.

**Table S3. Plasma and PET imaging ATN biomarkers in relation to the severity of cognitive impairment in Aβ- subjects (covariates: age, sex, education and *APOE* ε4).**

| *P* value | Overall | CDR = 0 *versus* CDR = 0.5 | CDR = 0 *versus* CDR ≥ 1 | CDR = 0.5  *versus* CDR ≥ 1 |
| --- | --- | --- | --- | --- |
| **PET biomarkers** |  |  |  |  |
| A: Global SUVR | 0.152 | 0.557 | 0.401 | 0.057 |
| T: MTL SUVR | 0.737 | 0.788 | 0.489 | 0.496 |
| T: NEO-T SUVR | 0.878 | 0.704 | 0.613 | 0.784 |
| N: metaROI SUVR | 0.057 | 0.836 | 0.075 | **0.020** |
| **Plasma biomarkers** |  |  |  |  |
| A: Aβ_42_/Aβ_40_ ratio | 0.062 | **0.019** | 0.070 | 0.784 |
| T: P-tau181, pg/mL | 0.359 | 0.174 | 0.197 | 0.828 |
| N: T-tau, pg/mL | 0.269 | 0.110 | 0.187 | 0.966 |
| N: NfL, pg/mL | **<0.001** | 0.872 | **0.005** | **<0.001**** |

Generalized linear models after adjustment for age, sex, education and *APOE* ε4 were applied to analyze the values of PET and plasma ATN biomarkers in relation to the severity of cognitive impairment. Unadjusted P values are presented for differences between the three CDR categories, those that reach significance (*P* < 0.05) before multiple comparisons adjustment are marked in bold, those that remain significant after multiple comparisons corrections (Bonferroni’s correction) are marked with asterisks (**, *P_c_* < 0.01). *APOE*, apolipoprotein E; CDR, Clinical Dementia Rating; PET, positron emission tomography; A/T/N, Amyloid/Tau/Neurodegeneration; SUVR, standardized uptake value ratio; SUVR, standardized uptake value ratio; MTL, medial temporal lobe; NEO-T, temporal neocortex; metaROI, meta-analytically derived region of interest; Aβ, β-amyloid; p-tau181, tau phosphorylated at threonine 181; t-tau, total tau; NfL, neurofilament light chain.

**Table S4. Plasma and PET imaging ATN biomarkers in relation to the severity of cognitive impairment in the entire cohort (covariates: age, sex, education and *APOE* ε4).**

| *P* value | Overall | CDR = 0  *versus* CDR = 0.5 | CDR = 0  *versus* CDR = 1 | CDR = 0  *versus* CDR ≥ 2 | CDR = 0.5 *versus* CDR = 1 | CDR = 0.5 *versus* CDR ≥ 2 | CDR = 1  *versus* CDR ≥ 2 |
| --- | --- | --- | --- | --- | --- | --- | --- |
| **PET biomarkers** |  |  |  |  |  |  |  |
| A: Global SUVR | **0.006** | 0.185 | **0.007** | 0.052 | **0.004** | 0.231 | 0.414 |
| T: MTL SUVR | **<0.001** | **0.049** | **<0.001*** | **<0.001***** | **<0.001*** | **<0.001***** | **0.002** |
| T: NEO-T SUVR | **<0.001** | **0.048** | **<0.001*** | **<0.001***** | **<0.001*** | **<0.001***** | **0.002** |
| N: metaROI SUVR | **<0.001** | 0.052 | **<0.001**** | **<0.001***** | **<0.001**** | **<0.001***** | **<0.001***** |
| **Plasma biomarkers** |  |  |  |  |  |  |  |
| A: Aβ_42_/Aβ_40_ ratio | 0.251 | 0.088 | 0.180 | 0.066 | 0.475 | 0.619 | 0.312 |
| T: P-tau181, pg/mL | **0.001** | **0.012** | **<0.001*** | **<0.001*** | **0.030** | 0.061 | 0.673 |
| N: T-tau, pg/mL | 0.490 | 0.249 | 0.136 | 0.204 | 0.453 | 0.702 | 0.899 |
| N: NfL, pg/mL | **<0.001** | 0.410 | **0.041** | **0.002** | **0.010** | **<0.001*** | **0.047** |

Generalized linear models after adjustment for age, sex, education and *APOE* ε4 were applied to analyze the values of PET and plasma ATN biomarkers in relation to the severity of cognitive impairment. Unadjusted P values are presented for differences between the four CDR categories, those that reach significance (*P* < 0.05) before multiple comparisons adjustment are marked in bold, those that remain significant after multiple comparisons corrections (Bonferroni’s correction) are marked with asterisks (***, *P_c_* < 0.001; **, *P_c_* < 0.01; *, *P_c_* < 0.05). *APOE*, apolipoprotein E; CDR, Clinical Dementia Rating; PET, positron emission tomography; A/T/N, Amyloid/Tau/Neurodegeneration; SUVR, standardized uptake value ratio; SUVR, standardized uptake value ratio; MTL, medial temporal lobe; NEO-T, temporal neocortex; metaROI, meta-analytically derived region of interest; Aβ, β-amyloid; p-tau181, tau phosphorylated at threonine 181; t-tau, total tau; NfL, neurofilament light chain.
